# Impairments in Pulmonary Function in Fontan Patients: Their Causes and Consequences

**DOI:** 10.3389/fped.2022.825841

**Published:** 2022-04-15

**Authors:** Karina Laohachai, Julian Ayer

**Affiliations:** ^1^Cardiology Department, Women's and Children's Hospital, Adelaide, SA, Australia; ^2^Faculty of Medicine and Health, The University of Sydney, Sydney, NSW, Australia; ^3^The Heart Centre for Children, Children's Hospital at Westmead, Sydney, NSW, Australia

**Keywords:** Fontan, pulmonary function, respiratory muscle, restrictive lung disease, diffusing capacity of carbon monoxide

## Abstract

Patients with a Fontan circulation lack a sub-pulmonary ventricle with pulmonary blood flow passively redirected to the lungs. In the Fontan circulation, ventilation has a significant influence on pulmonary blood flow and cardiac output both at rest and with exercise. Children and adults with a Fontan circulation have abnormalities in lung function. In particular, restrictive ventilatory patterns, as measured by spirometry, and impaired gas transfer, as measured by the diffusing capacity of carbon monoxide, have been frequently observed. These abnormalities in lung function are associated with reduced exercise capacity and quality of life. Moderate to severe impairment in lung volumes is independently associated with reduced survival in adults with congenital heart disease. Skeletal and inspiratory muscle weakness has also been reported in patients with a Fontan circulation, with the prospect of improving respiratory muscle function through exercise training programs. In this review, we will present data on cardiopulmonary interactions in the Fontan circulation, the prevalence and severity of impaired lung function, and respiratory muscle function in this population. We will discuss potential causes for and consequence of respiratory impairments, and their impact on exercise capacity and longer-term Fontan outcome. We aim to shed light on possible strategies to reduce morbidity by improving respiratory function in this growing population of patients.

## Introduction

Children born with univentricular anatomy undergo procedures in early life resulting in the Fontan circulation. This is routinely performed as staged procedures, resulting in systemic venous return from the superior and inferior vena cava (SVC and IVC) draining passively into the pulmonary arteries, bypassing a sub-pulmonary pump. The completion of the Fontan circuit reduces desaturation and unloads the functionally single ventricle. Heart rate, ventricular function, respiration, and skeletal and respiratory muscle strength all affect the performance of the Fontan circulation.

Since its initial description, the Fontan procedure has undergone several modifications to improve the circulation's efficiency and reduce overall morbidity and mortality. However, morbidity in this population is still high. The Fontan circulation results in high systemic venous pressure, chronic venous congestion, and reduced pulmonary blood flow, cardiac output, and ventricular function. In addition, multiple other adverse factors may be present including chronotropic incompetence, non-uniform distribution of pulmonary blood flow, chest wall and spinal deformities, pleural adhesions, diaphragmatic palsy, and respiratory and skeletal muscle weakness. With advances in surgical techniques and medical therapy, there are an increasing number of Fontan patients surviving to older adulthood. However, complications related to the Fontan circulation are common, including ventricular systolic and diastolic dysfunction, arrhythmia, venous thrombosis, protein-losing enteropathy, plastic bronchitis, ascites, and hepatic fibrosis and carcinoma (1, 2). Approximately 50% of patients require another intervention by 15 years after Fontan completion (3, 4), and freedom from Fontan failure (defined as occurrence of death, protein-losing enteropathy, plastic bronchitis, poor functional class, or heart transplant) at 50 years of age is only 30% (5).

Abnormal lung function and respiratory muscle weakness have been documented in Fontan patients. Moderate to severe impairment in lung volumes are independently associated with reduced survival in adults with congenital heart disease (6). Fontan patients have reduced total lung capacity and vital capacity, with a restrictive ventilatory pattern (7, 8). These impairments of lung function are associated with reduced exercise capacity (9, 10). For these reasons, treatments directed at improving lung function both before and after Fontan completion are of great interest. Further advances in medical care are actively being sought to reduce late morbidity and mortality after Fontan completion. As yet, very few medical therapies have been shown to be effective. Cardiopulmonary rehabilitation has been shown to improve surrogate outcome measures in patients with a Fontan circulation such as aerobic capacity.

## Cardiopulmonary Interactions in the Normal Circulation

Respiration induces changes in intrathoracic pressures and thereby lung volumes, which in turn affects preload, afterload, and stroke volume. Lauson et al. described the influence of respiration on changes in circulatory pressure in normal subjects, those with chronic lung disease, and those with rheumatic heart disease (11). They found only small variations in pressures during tidal breathing consistent with minimal changes in heart rate or stroke volume. However, during deep inspiration, they found an increase in systemic venous return and right ventricular (RV) stroke volume, and a decrease in left ventricular (LV) stroke volume. In the normal circulation, the majority of LV filling occurs during the first portion of ventricular diastole with atrial systole providing a small contribution at end diastole. Using electrocardiographic and respiratory gated echocardiography, Riggs and Snider demonstrated that the reduction in LV stroke volume during inspiration is secondary to a reduction in early LV filling and not during atrial systole (12, 13). Heart rate also increases with inspiration (12). Several mechanisms for these effects of inspiration have been proposed: (1) an increase in pulmonary venous capacitance, (2) reduced diastolic filling time secondary to the accelerated heart rate, (3) an increase in systemic afterload secondary to increase in intrathoracic pressure, and (4) alterations in the interventricular septal shape secondary to RV filling resulting in increased LV diastolic pressure (12, 14).

Systemic venous return from the SVC and IVC are dependent on the existence of a pressure gradient between the extra-thoracic venous system and the right atrium (15). The venous system is a low-resistance, low-pressure, and high-compliance circulation. During inspiration, a decrease in intra-pleural pressure occurs from contraction of the external intercostal and diaphragmatic muscles, causing an increase in right atrial transmural pressure (pressure exerted across the wall) (11). This results in the right atrial chamber distending and a reduction in right atrial pressure, increasing systemic venous return. With contraction of the diaphragmatic muscles during inspiration, intra-abdominal pressure increases, and transmural pressure of the abdominal vessels decreases. This effectively causes constriction of the abdominal vessels, increasing IVC return to the right atrium. Once right atrial pressure increases, systemic venous return decreases (16). Through an adrenergic response, veno-constriction occurs, increasing vascular pressure and maintaining venous return. In addition to this, the renin–angiotensin–aldosterone system is activated, increasing reabsorption of water and sodium back into the circulation, vasoconstricting arterioles, and releasing anti-diuretic hormone.

Riggs and Snider also noted an increase in RV filling during inspiration, in both early and late diastole (12). Compared with LV filling, they found a greater portion of RV filling occurring during atrial systole. This may be augmented by the diaphragm descending and transiently compressing compliant hepatic sinusoids and portal venules (17). In summary, in the normal circulation, inspiration is associated with increased RV and reduced LV stroke volume.

## Cardiopulmonary Interactions in the Fontan Circulation

In the Fontan circulation, systemic venous return is independent of a ventricular pump and relies on a balance between systemic and pulmonary vascular resistance (PVR). The initial belief was that a contractile chamber (right atrium) acting as a pump to drive pulmonary forward flow was important in the Fontan circulation (18). Subsequent animal studies, however, demonstrated that right atrial contraction had a limited role in actively pumping blood forward against higher resistance in the pulmonary arteries (19, 20). de Leval et al. examining Fontan hemodynamics *in vitro*, demonstrated that a contractile chamber did not improve forward flow and did in fact limit pulmonary forward flow detrimental to the circulation by increasing upstream resistance (21).

The importance of spontaneous respiration in the Fontan circulation was highlighted by Fontan and Baudet in 1971, stating that “respiratory assistance should be stopped early because positive pressure prevents central venous return” (18). Subsequent studies have shown respiration to be an important factor, with inspiration augmenting antegrade blood flow into the pulmonary arteries, through inducing negative intrathoracic pressure (22, 23). Spontaneous breathing has been shown to be the main determinant of cardiac output in Fontan patients (23, 24). Penny and Redington demonstrated that inspiration augments antegrade pulmonary blood flow in an atriopulmonary Fontan circulation, with pulmonary forward flow nearly 64% higher during inspiration than expiration (22). Antegrade pulmonary blood flow and peak velocity increased during atrial systole, and was further augmented with inspiration (22).

Redington et al. also demonstrated augmentation of pulmonary blood flow during inspiration, and attenuation or cessation of this antegrade flow at beginning of expiration in the Fontan circulation (23). They found that during normal tidal breathing, the cardiac cycle had no significant effect on pulmonary blood flow. Through performing breathing maneuvers, they could demonstrate an increase in antegrade pulmonary flow during prolonged forced inspiration against a closed glottis (Mueller maneuver), and cessation to flow with forced expiration (Valsalva), apart from some low-velocity pulsatile flow during ventricular systole.

Consistent with previous studies demonstrating an increase in venous return to the RV, Hsia et al. confirmed higher forward flow in the hepatic veins and IVC during inspiration in both normal and Fontan subjects (24). In normal subjects, there was biphasic forward flow within the hepatic veins and IVC, with a small amount of reversal during atrial systole. This normal pattern was absent in Fontan subjects where the atrium was excluded from the circulation. Thirty percent of flow through the Fontan conduit was dependent on respiration, compared with 15% in normal subjects. Approximately 55% of hepatic flow was respiratory dependent. They postulated that this was secondary to hepatic venous congestion and reduced compressibility. They also found that gravity had a more significant hemodynamic effect on Fontan than normal subjects, with reduced net forward flow and increased flow reversal when erect (24). Shafer et al. also demonstrated the effects of expiration in Fontan subjects during exercise, with a reduction in stroke volume during exercise with an expiratory load (25).

**Figure 1:**
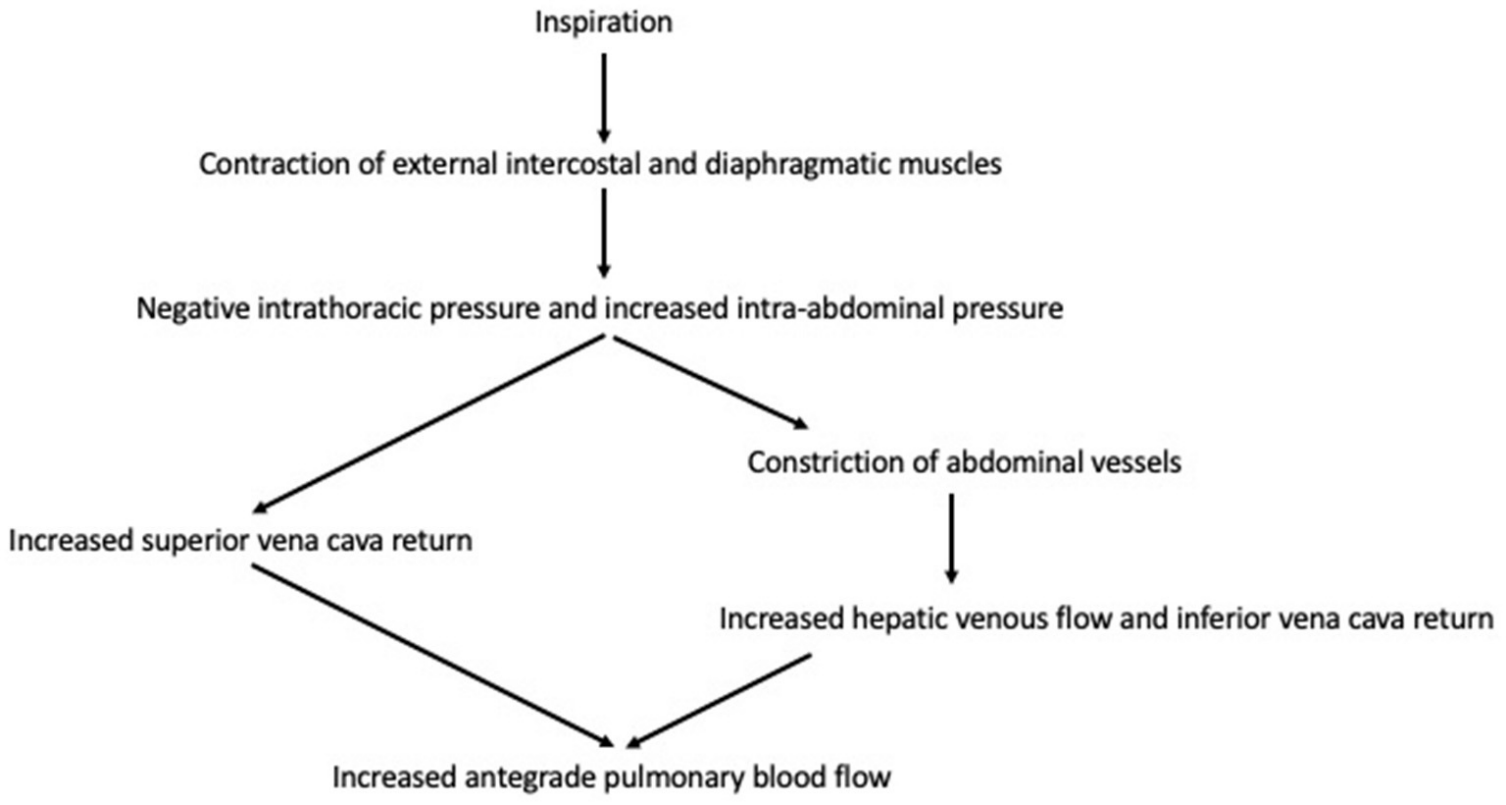
Cardiopulmonary interactions in Fontan circulation.

In summary, these studies demonstrate the alterations from normal in cardiopulmonary interactions in the Fontan circulation (Figure 1). The effects of respiration are more pronounced, with inspiration augmenting pulmonary antegrade flow. These changes are induced by changes in intrathoracic and intra-abdominal pressures, thereby influencing SVC and IVC return.

## Role of MRI in Assessment of Respiratory Effects of Pulmonary Blood Flow at Rest and With Exercise

Using phase-contrast techniques, cardiac magnetic resonance imaging (CMR) provides a unique opportunity to study cardiopulmonary interactions of the Fontan circulation. Respiratory variability in flows through the Fontan circulation has been shown with CMR, with increased flow rates during inspiration (26–29). For example, Hjortdal et al. examined the effects of breathing on flow during exercise (26). Inspiratory time initially increased during exercise. At rest, mean aortic flow was marginally lower during inspiration than expiration and was more dependent on the cardiac cycle than respiration. Stroke volume was also lower during inspiration, and this was unchanged with exercise. In contrast, IVC flow was higher during inspiration at rest, with a smaller differential during exercise. They found no respiratory dependence on SVC flow, but noted limitations in their real-time flow MRI technique.

Using real-time free breathing methods, Wei et al. measured respiration and flow in Fontan subjects during rest and exercise (27). They also demonstrated increased IVC flow with inspiration, which persisted with exercise, and additionally showed increased SVC flow during inspiration. However, there was no significant change in total systemic venous flow between breath holding and free breathing, which could be accounted for by the exaggeration of reduced or even reversed flow during expiration. They also demonstrated a significant increase in aortic, SVC, and IVC flows with exercise. These findings are in keeping with Hjortdal et al. apart from the increase in SVC flow with exercise. This difference may be explained by differences in real-time flow techniques and a younger cohort in Wei's et al. study population (12.4 ± 4.6 years compared with 20.0 ± 6.3 years) (26, 27).

Significant increases in systemic venous and aortic flow pulsatility with respiration have been shown, with variability between vessels (26, 30). Gabbert et al. developed a detailed matrix to assess venous flow hemodynamics in the Fontan circulation, to quantify respiratory and cardiac dependence of flow (30). They found that although respiration had a significant effect on systemic venous flow pulsatility, the predominant determinant of IVC flow was the heart and not the lungs. These findings were supported by Fogel et al. who showed that ~70% of systemic venous flow at rest was cardiac dependent, with the highest flow occurring at end-systole and early-diastole (28). Like others, Fogel et al. also showed increased flow during inspiration at the mid-conduit level.

In a small cohort, where we were examining the hemodynamic responses to inspiratory muscle training (IMT), we used CMR to assess aortic and pulmonary flow at rest and during exercise (31). We found that both stroke volume and ejection fraction increased with exercise. We also demonstrated increased pulmonary flow during inspiration at rest. Although IMT improved inspiratory muscle strength and ventilatory efficiency on exercise testing, aortic and pulmonary forward flow did not change.

CMR has further evaluated changes in pulmonary blood flow during exercise in the Fontan circulation. Changes in pulmonary forward flow are both cardiac and respiratory dependent. Systemic venous return, particularly IVC return, and pulmonary antegrade flow increases during inspiration at rest.

## Determinants of Cardiac Output in the Fontan Circulation

In the normal circulation, cardiac output is a function of stroke volume (itself influenced by contractility, preload, and afterload) and heart rate. Several factors can potentially limit cardiac output in the Fontan circulation. Primarily, the lack of a sub-pulmonary ventricle results in preload insufficiency (32, 33) with consequent adverse ventricular remodeling (34, 35) and impaired diastolic ventricular filling (36, 37). As a result of this preload insufficiency, increasing heart rate may have a blunted ability to increase cardiac output in the Fontan circulation (38). Other factors likely to impact cardiac output in the Fontan circulation include systolic dysfunction (34, 39), increased systemic afterload (40), and abnormal ventriculo-arterial coupling (41).

Numerous studies have shown the importance of PVR in the Fontan circulation and is now thought to be the major determinant of cardiac output (42, 43). As the systemic and pulmonary circuits in the Fontan circulation are connected without a pump in between, stroke volume is dependent on pulmonary venous return (preload), which in turn is dependent on PVR. Gewillig and Goldberg demonstrated changes in cardiac output with alterations in ventricular systolic function and PVR (44). Mild increases in PVR cause significant reduction in cardiac output. Factors that can influence PVR include patency of the Fontan circuit, branch pulmonary arteries, pulmonary capillary bed and pulmonary veins, and respiratory function.

Pulmonary vasculature development is abnormal in patients with complex congenital heart. Antenatal hemodynamic factors can alter pulmonary artery size and arborization, and pulmonary venous and lymphatic anatomy. The connection between the cardiac and pulmonary circulations is evident embryologically with lung endoderm protruding into the mesoderm as the heart tube elongates and folds (45). By 20 weeks of gestation, pre-acinar pulmonary arteries have already formed, and any mal-development of cardiac structure has already occurred. Therefore, hemodynamic changes within the circulation, such as a restrictive atrial septum in hypoplastic left heart syndrome, can adversely affect pulmonary artery development. Lack of pulsatile pulmonary flow in the Fontan circulation has been shown to be associated with endothelial dysfunction and abnormal vascular development (46, 47). This has also been shown on histological specimens, with Levy et al. documenting in a study of lung biopsies from 18 Fontan patients, variable intimal proliferation and muscularization of terminal bronchiole and alveolar duct arteries (48). Whether abnormalities of pulmonary vascular development can impact alveolar development remains to be determined.

Augmentation of cardiac output is usually achieved by increases in heart rate, preload and/or myocardial contractility, and reduced afterload. With exercise, for example, biventricular stroke volume increases in the setting of adequate preload reserve (43). In the Fontan circulation, ventricular function does not predict exercise performance, suggesting that it is not the main limitation of exercise capacity (49). The sub-pulmonary ventricle has now been shown to also play a significant role during exercise in the normal circulation (50, 51). La Gerche and Gewillig elegantly discussed the effects of increased RV afterload, and reducing LV preload and consequently cardiac output; they further proposed that without a sub-pulmonary pumping chamber in the Fontan circulation, PVR becomes the limiting factor of cardiac output limitation (51).

## Acute Changes in Pulmonary Vascular Resistance and Positive Pressure Ventilation in the Fontan Circulation

Pulmonary blood flow and PVR are the major determinants of cardiac output in the Fontan circuit and are significantly affected by respiration. In a normal circulation, PVR is the main determinant of pulmonary afterload and is affected by lung volumes (52). Reduction in intrathoracic pressure with inspiration aids antegrade pulmonary blood flow and consequently cardiac output.

Positive pressure ventilation (PPV) is frequently used in the management of respiratory failure in postoperative cardiac patients. However, it has been shown to reduce cardiac output likely secondary to increases in PVR and reduced systemic venous return. The hemodynamics of a Fontan circulation are dependent on low PVR and sufficient venous return, and hence the adverse effect of PPV is augmented in the Fontan circulation (53). PPV can be used to increase pressures during inspiration (IPPV) and expiration (PEEP), resulting in increased intrathoracic pressures. Applying pressure during expiration prevents intrathoracic pressures from returning to normal, thereby limiting systemic venous return and cardiac output (54–56). Cournand and Motley showed that reduction in cardiac output was inversely proportional to the pressure delivered (57). This reduction in cardiac output is exaggerated in the Fontan circuit, also reducing antegrade flow from the SVC into the branch pulmonary arteries. These findings highlight the importance of early extubation and/or minimizing positive pressure post-operatively after Fontan completion (23, 58). Jardin et al. proposed that in otherwise healthy patients with respiratory distress syndrome, leftward displacement of the interventricular septum limits LV filling and cardiac output (56). Ventricular–ventricular interaction is also present in Fontan patients with Fogel et al. demonstrating marked differences in the wall motion of systemic right ventricles depending on the presence of a left ventricle, through a magnetic resonance tagging technique (59). They concluded that this ventricular–ventricular interaction plays a significant role in the mechanisms of the systemic ventricle in the Fontan circulation. However, the role of respiratory-dependent septal shift in “biventricular” Fontan patients (i.e., those with two ventricles present) is unknown.

Since the nineteenth century, negative pressure ventilation (NPV) has been used in paralyzed patients with respiratory insufficiency. It has been shown to improve both pulmonary blood flow and cardiac output, particularly in the Fontan circulation, and is associated with improved systemic venous return (15, 60–63). NPV applies sub-atmospheric pressures to the thorax during inspiration, causing the thorax to expand, thereby reducing alveolar pressures, lowering PVR, and augmenting systemic venous return (64).

Shekerdemian et al. converted patients in intensive care after Fontan completion from PPV to NPV and demonstrated acute improvement in pulmonary antegrade flow (15). This was associated with improved mixed venous saturations and increasing cardiac output, without changes in heart rate, by over 50% during both the acute and late post-operative periods (15), highlighting the importance of the respiratory system in the post-operative Fontan circulation. Charla et al. also examined the role of 10 min of NPV and biphasic ventilation (BPV) in the ambulatory Fontan population (65). Using CMR, they also found baseline low pulmonary blood flow compared with controls. With both NPV and BPV, there was a significant improvement in both pulmonary blood flow and cardiac output compared with controls. This is most likely secondary to changes in intrathoracic pressures, as previously demonstrated with normal inspiration (22). They saw a greater improvement with BPV, which was postulated as being due to BPV supporting both the inspiratory phase and maintaining intra-abdominal pressures during expiration, thereby minimizing retrograde flow (65). This was supported by their demonstration of increased IVC and hepatic venous flows. In addition, subjects tolerated short-term external ventilation well and their willingness to continue external ventilation correlated with the improvement in pulmonary blood flow (65). These studies examining NPV in Fontan patients have assessed acute hemodynamic response only. Long-term safety, tolerability, and efficacy of NPV remain an interesting area for future research.

## Restrictive Lung Disease

### Reduced Lung Volumes

Lung volume, alveolar surface area, and number increases from 29 weeks' gestation to at least 12 weeks postnatally, with a close association to body weight (66). During the first 3 years of life, increases in lung volume are predominantly due to increases in alveolar number rather than size. Subsequently, alveoli increase in both number and size, continuing through childhood and into adolescence, although at a reduced rate, with 95% of alveolar surface area in adults being formed after birth (67). Lung development is altered, with abnormal lung parenchyma and pulmonary vasculature, in subjects with congenital heart disease, even in the absence of medical procedures and surgical intervention (68).

Prior to Fontan completion, neonatal palliation of pulmonary blood flow may result in abnormalities of pulmonary vascular development. After Fontan completion, pulmonary blood flow is non-pulsatile with altered wall shear stress and reduced pulmonary endothelial function (46). The degree to which alveolar development is impacted by abnormal pulmonary vascular development in patients with a Fontan circulation remains to be determined. Lung development may also be adversely impacted by other factors commonly seen in patients with congenital heart disease: desaturation, mechanical ventilation, lymphatic dysfunction, multiple sternotomies and thoracotomies, scoliosis or pectus deformity, and postoperative complications such as pleural adhesions and diaphragmatic palsy. Diaphragmatic palsy, for example, can reduce ventilatory function by ~25%, particularly in the setting of generalized respiratory muscle weakness (69).

Animal studies have demonstrated abnormal lung parenchyma after Fontan completion (70). Kanakis et al. (70) demonstrated normal lung parenchyma in 8 pigs at baseline, with rapid development of a mononuclear infiltration, in keeping with bronchiolitis, within 2 h of Fontan completion. Pulmonary capillary recruitment has also been demonstrated in isolated dog lungs, with rises in pulmonary arterial pressure and pulsatile flow (71). This finding suggests that long-term non-pulsatile flow, in the setting of Fontan circulation, causes adverse parenchyma lung remodeling and increases in PVR. Numerous studies have shown reduced total lung capacity and vital capacity in Fontan patients, suggestive of small lungs (7, 8, 72–77). The prevalence of restrictive lung disease is high at 58–60%, with all studies finding reduced forced expiratory volume in 1 s (FEV_1_), forced vital capacity (FVC), and a normal or high FEV_1_/FVC ratio (9, 10, 77). Ohuchi et al. found reduced total lung capacity (TLC), vital capacity, and functional residual capacity in Fontan subjects compared with controls (8). Although their Fontan cohort had normal residual volumes, RV/TLC ratio was increased indicative of air trapping. These authors speculated that repeated surgical interventions lead to reduced mechanical mobility of the lungs, causing air trapping (8). Matthews et al. also found an increased RV/TLC ratio but an increased residual volume when measured by plethysmography (Z-score 2.46 ± 1.87) compared with the standard helium dilution single breath test used in other studies (72). They speculated that the difference between methods was due to the single breath test only measuring gas communicating with large airways.

In a large cohort of Fontan subjects aged 6–18 years from the Pediatric Heart Network Fontan Cross-Sectional study, Opotowsky et al. found a high percentage (45.8%) of subjects with low FVC (76). This low FVC was not associated with any demographic or clinical variable. Guenette et al. found significantly reduced FEV_1_ and FVC in their Fontan cohort compared with controls, with 65% having a restrictive pattern (78). Moderate restriction was identified in 44% of subjects studied by Turquetto et al., which was associated with presence of postural deviations (e.g. kyphosis and scoliosis) and previous thoracotomies (9). Restrictive lung disease has also been associated with number of interventions, low body mass index, scoliosis, and diaphragmatic paralysis (10).

Ohuchi et al. found that vital capacity was associated with the number of other previous surgical procedures performed (average of 1–2.4 procedures) and demonstrated that during follow-up (0.7–17.5 years), vital capacity decreased significantly (73). The significance of the restrictive lung disease is highlighted by Callegari et al. finding a correlation between FEV_1_ % predicted and self-reported quality of life scores, related to physical functioining (10).

### Ventilatory Limitation to Exercise in Patients With a Fontan Circulation

In Fontan subjects, reduced FVC is associated with low peak oxygen consumption (VO_2_) and reduced exercise capacity, and is a predictor of survival in adults with congenital heart disease (6, 79). In adults with congenital heart disease, presence of restrictive lung disease is a strong predictor of exercise capacity (79). It is now recognized that a significant proportion of patients with a Fontan circulation have ventilatory rather than circulatory limitations to exercise capacity (10, 76, 78).

Guenette et al. reported higher activity-related dyspnea in their Fontan cohort compared with controls (78). In keeping with previous studies, Guenette et al. also found significantly reduced FEV_1_, FVC, maximal voluntary ventilation, and diffusing capacity of carbon monoxide (DLCO), with a restrictive ventilatory pattern (78). They examined the cardiopulmonary response to exercise in Fontan subjects, noting significantly reduced peak minute ventilation (VE) compared with controls, secondary to reduced peak tidal volume. Fontan subjects adopted a more rapid breathing pattern at any given exercise intensity. To further characterize ventilatory limitation to exercise, these authors performed inspiratory flow-volume loops during exercise. This demonstrated a higher end inspiratory lung volume, indicative of reduced inspiratory reserve volume. There was no evidence of dynamic lung hyperinflation. Ventilatory equivalence of carbon dioxide (VE/VCO_2_) slope, a marker of ventilatory efficiency, during exercise was significantly elevated. Their findings suggest that the restrictive pattern of lung function in Fontan subjects contributes to an abnormal ventilatory response to exercise. Previous studies have also shown elevated ventilatory equivalence of oxygen (VE/VO_2_) at both rest and exercise in functionally single ventricle patients (80) Like other studies (9, 10), Turquetto et al. also found a strong correlation between peak VO_2_ and lung function parameters (FEV_1_, FVC, total lung capacity, and diffusing capacity of carbon monoxide) (9).

Impairments in pulmonary function can impact exercise capacity, with Opotowsky et al. demonstrating that a low FVC was predictive of low peak VO_2_, and a stronger determinant than ventricular morphology or dysfunction (76). They also showed that Fontan subjects with an elevated VE/VCO_2_ slope were more likely to have a low breathing reserve (BR), suggestive of ventilatory limitation of exercise. In comparing those with ventilatory limitation of exercise (defined as BR <20%) and those with presumed cardiac limitation (BR > 20%), low FVC, high VE/VCO_2_ slope, and high body mass index independently predicted ventilatory limitation.

## Reduced DLCO

DLCO is a measure of lung gas transfer from alveolar gas to hemoglobin within the pulmonary capillaries. It is affected by diffusion across the alveolar-capillary membrane, hemoglobin levels, and capillary blood volume. Therefore, reduced DLCO may be secondary to reduced capillary blood volume available for gas transfer, reduced alveolar volume, and/or abnormal alveolar membrane conductance. DLCO is strongly associated with aerobic capacity, measured by peak VO_2_ (81).

Fontan patients have reduced DLCO, ranging from Z-scores of −2.85 to −3.1 (7–9, 72, 74). Matthews et al. proposed that the reduction in DLCO may be due to two mechanisms: (1) the non-pulsatile nature of the pulmonary blood flow inducing changes within the pulmonary bed and causing thickening of the alveolar capillary membrane; (2) recurrent microembolism (72). Larsson et al. also proposed that the non-pulsatile nature of pulmonary blood flow impairs gas exchange within the lungs (7). This is in keeping with Levy et al. who demonstrated thick-walled distal pulmonary arteries, with wall thickness correlating to outcomes (48). Idorn et al. examined the etiology of reduced DLCO in more detail through assessing different components of DLCO (74). In their cohort, they found reduced pulmonary capillary blood volume but normal diffusing capacity across the alveolar membrane. Their data suggested preserved alveolar membrane function and reduced pulmonary perfusion. These authors also found an increase in DLCO and increased capillary blood volume in the supine compared with sitting position. They speculated that this may be secondary to improved perfusion of the upper lobes, secondary to blood flow being more gravity dependent in the absence of a sub-pulmonary pump. In a small cohort of 19 Fontan patients, del Torso et al. also found abnormalities in lung perfusion in 8 of their 19 patients, with majority being localized perfusion defects (82). Matsushita et al. went on to demonstrate normal ventilation in Fontan patients (83) and like others showed gravity-dependent blood redistribution (84, 85).

Further studies potentially utilizing double diffusion (DLCO and the diffusing capacity of nitric oxide, DLNO) are required to determine the exact etiology of low DLCO in the Fontan population.

## Respiratory Muscle Weakness in Patients With Congenital Heart Disease

Greutmann et al. have documented generalized muscle weakness involving the skeletal and respiratory muscles in patients with congenital heart disease (86). They studied 41 subjects with congenital heart disease, including 11 subjects with single ventricle physiology. Maximal inspiratory pressure (MIP) was significantly reduced in the congenital heart disease group, measuring 75 ± 26 cmH_2_O (77±27%) compared with 102 ± 32 cmH_2_O in the control group. Inspiratory muscle weakness was greater than expiratory muscle weakness. MIP and maximal expiratory pressure (MEP) correlated significantly with peak VO_2_. In addition, subjects with globally reduced respiratory muscle strength (both MIP and MEP) had lower maximal voluntary minute ventilation, which at peak exercise was also associated with peak VO_2_. Similarly, Turquetto et al. examined respiratory muscle strength in 27 Fontan patients (9) by using two non-invasive modalities, MIP and sniff nasal inspiratory pressure (SNIP). They also found reduced muscle strength, measuring MIPs of 76 ± 23 cmH_2_O (63 ± 16% predicted) in males and 81 ± 33 cmH_2_O (71 ± 32% predicted) in females. SNIP was measured at 99 ± 24 cmH_2_O (72 ± 31%) in males and 84 ± 13 cmH_2_O (82 ± 12%) in females. Furthermore, they found an association between SNIP and peak VO_2_. This respiratory muscle weakness may contribute to reduced lung volumes, as seen in Fontan patients.

## Improving Fontan Outcomes by Improving Pulmonary Function

With the identification of pulmonary abnormalities in Fontan patients, research studies are now determining ways to improve lung function, with the hope to improve exercise capacity, quality of life, and morbidity. With the identification of reduced skeletal and respiratory muscle strength, and better understanding of altered cardiopulmonary interactions in the Fontan circulation, mechanisms to improve lung function have been proposed.

### Respiratory Muscle Training

Like skeletal muscles, respiratory muscles can be trained with regular pressure loading. Respiratory muscle weakness affects exercise capacity by predisposing them to fatigue and an increased perception of dyspnea (87). During maximal exercise, 14–16% of cardiac output supplies the respiratory muscles (88). It has been speculated that strengthening respiratory muscles can augment skeletal blood flow by reducing blood diverted to the respiratory muscles.

IMT has been studied in a number of conditions including chronic heart failure (89–91). It has been shown to improve exercise capacity through strengthening of the inspiratory muscles and attenuating the exaggerated peripheral vasoconstriction in exercising limbs (92).

We were the first to show that 6 weeks of IMT improved inspiratory muscle strength (measured by MIP), ventilatory efficiency of exercise (measured by VE/VCO_2_), and resting cardiac output in young Fontan patients (31). This was performed with patients training for 30 min a day at 30% of individual MIP, with an increase in threshold at 3 weeks. MIP improved from 69 ± 22 cmH_2_O to 103 ± 32 cmH_2_O, which is more comparable with MIP found in healthy controls (9). In a subsequent pilot study, Wu et al. assessed the effects of 12 weeks of IMT for 30 min a day at 40% of their initial measured MIP. Although they found no improvement in MIP, there was an improvement in peak work rate, and a trend toward improved peak VO_2_ and ventilatory efficiency (93). Fritz et al. looked at a longer 6-month period of IMT, with subjects training for 10–30 repetitions a day and a load that was increased at the patient's discretion. They found improvement in resting oxygen saturations, indicative of improvement in ventilation/perfusion matching, but no improvement in lung function or exercise capacity (94). They proposed that the differences in study findings are secondary to different IMT regimes.

More recently, Turquetto et al. performed a randomized controlled trial looking at combined IMT and aerobic training (95). Fontan subjects were randomized to either personalized aerobic training or IMT, and a non-exercise group was used as a control. Their IMT regime consisted of 3 sets of 30 repetitions at 60% of individual MIP for 4 months, with adjustment in load throughout the duration. Aerobic training consisted of 60-min supervised, individually prescribed exercise training (treadmill, light resistance, and stretching) 3 times a week for 4 months. They found an improvement in peak VO_2_ with both these training regimes, but a higher improvement in the aerobic training group.

Ait Ali et al. explored this concept further through the assessment of controlled breathing (respiratory training) in Fontan patients, training both the inspiratory and expiratory muscles (96). They used a method that forms the basis of a yoga practice, involving conscious diaphragmatic contraction and relaxation. This involved weekly 2-h sessions for 3 months of respiratory training. Diaphragmatic respiration increased intrathoracic negative pressure, optimizing systemic venous return. They demonstrated improvement in peak VO_2_ and endurance time.

These studies are suggestive that with the correct training regime, including adjustments of the load according to the patient's individual MIP, both IMT and respiratory training have the potential to improve exercise capacity. However, larger studies need to be undertaken to determine the potential benefits, particularly in the setting of other physical activity programs. IMT is a simple and safe intervention that can be used to improve respiratory muscle strength.

### Resistance and Endurance Training

Due to the effects of gravity, when exercising, peripheral skeletal muscles need to pump against gravity to maintain diastolic filling. In the Fontan circulation, pulmonary blood flow is dependent on IVC return. Lower limb venous compliance correlated with calf surface area and muscle mass (97). Cordina et al. therefore hypothesized that enhancement of lower limb muscle mass could improve cardiac output (98). They achieved this through a 20-week gym-training program where they found that isolated muscle resistance training improved exercise capacity.

Hedlund et al. examined the effects of endurance training on Fontan patients, through individualized endurance training programs, including supervised weekly training sessions for 45 min over the duration of 12 weeks (75). These programs consisted of a variety of sports running, dancing, cycling, and swimming. They reported increases in vital capacity after 3 months of training, which was not observed in their control group. In a separate study, they also found improved quality of life, as reported by the Fontan patients and their parents, highlighting the importance of some form of exercise in this population (99). The benefits on lung function of aerobic and/or resistive exercise programs in patients with a Fontan circulation remain to be tested.

## Conclusion

Patients with a Fontan circulation have abnormal cardiopulmonary interaction, in the setting of their systemic and pulmonary circulations being in series. Due to this, the physiological changes with inspiration are more pronounced in subjects with a Fontan circulation, and respiration has a more crucial role in regulating cardiac output. The critical dependence of ventilation in determining cardiac output in the Fontan circulation is highlighted by the acute use of NPV or BPV immediately post-Fontan completion to improve antegrade pulmonary blood flow and cardiac output. Fontan patients have been shown to have reduced lung volumes with restrictive lung disease, and abnormal lung gas transfer. The exact etiology of these changes still needs to be studied in more detail. In addition, Fontan patients have reduced skeletal and respiratory muscle strength, further limiting exercise capacity. We and others have shown potential benefits to respiratory muscle training. The benefits of aerobic and skeletal muscle training with or without respiratory muscle training remain to be determined.

## Author Contributions

KL and JA wrote and revised the article. Both authors contributed to the article and approved the submitted version.

## Conflict of Interest

The authors declare that the research was conducted in the absence of any commercial or financial relationships that could be construed as a potential conflict of interest.

## Publisher's Note

All claims expressed in this article are solely those of the authors and do not necessarily represent those of their affiliated organizations, or those of the publisher, the editors and the reviewers. Any product that may be evaluated in this article, or claim that may be made by its manufacturer, is not guaranteed or endorsed by the publisher.
